# Implementation of a parentage control system in Portuguese beef-cattle with a panel of microsatellite markers

**DOI:** 10.1590/S1415-47572009005000026

**Published:** 2009-03-06

**Authors:** Inês Carolino, Conceição O. Sousa, Sónia Ferreira, Nuno Carolino, Fátima S. Silva, Luís T. Gama

**Affiliations:** 1 Unidade de Investigação de Recursos Genéticos, Reprodução e Melhoramento Animal, Instituto Nacional de Recursos Biológicos, Vale de SantarémPortugal; 2 Instituto Superior de Agronomia, Tapada da Ajuda, LisboaPortugal; 3 Faculdade de Medicina Veterinária, Universidade Técnica de Lisboa, LisboaPortugal

**Keywords:** cattle, genetic markers, microsatellites, parentage control

## Abstract

A study was conducted to assess the feasibility of applying a panel of 10 microsatellite markers in parentage control of beef cattle in Portugal. In the first stage, DNA samples were collected from 475 randomly selected animals of the Charolais, Limousin and Preta breeds. Across breeds and genetic markers, means for average number of alleles, effective number of alleles, expected heterozygosity and polymorphic information content, were 8.20, 4.43, 0.733 and 0.70, respectively. Enlightenment from the various markers differed among breeds, but the set of 10 markers resulted in a combined probability above 0.9995 in the ability to exclude a random putative parent. The marker-set thus developed was later used for parentage control in a group of 140 calves from several breeds, where there was the suspicion of possible faulty parentage recording. Overall, 76.4% of the calves in this group were compatible with the recorded parents, with most incompatibilities due to misidentification of the dam. Efforts must be made to improve the quality of pedigree information, with particular emphasis on information recorded at the calf's birth.

Pedigree recording is an essential step in conservation and selection programs in most livestock breeds. When conservation is the major concern, the objective is often to control inbreeding by preventing the breeding of closely related individuals, in order to minimize the associated loss of genetic variability and fitness. On the other hand, pedigree information is also of crucial importance in selection programs, because family information is often considered in selection decisions. In recent years, mixed model methods have been widely used for genetic evaluation in different livestock species, usually with an animal model, whereby information on all relatives of an individual is taken into account when predicting breeding values (Henderson, 1984). Therefore, a major concern in this case is the reliability of pedigree information, as pedigree errors may reduce the accuracy of selection, and thus hamper genetic progress (Van Vleck, 1970, Gelderman *et al.*, 1986, Visscher *et al.*, 2002). Banos *et al.* (2001), on simulating a dairy-cattle selection nucleus, found that an assumed rate of paternity misidentification of 11% would result in a decrease of 11 to 15% in the genetic trend for milk-traits.

In spite of the importance of pedigree information in breeding programs, its recording is not an easy task, especially in breeds produced under extensive conditions, mostly because of the costs involved in mating control and registration of offspring at birth. Therefore, cost-effective parentage control systems that can be implemented under common production conditions are of capital importance for both conservation and improvement programs in livestock.

In Portugal, beef production is largely based on native breeds and their crosses with exotic germplasm, of which Charolais and Limousin are the most common sire-breeds in crossbreeding programs. Cows are usually pasture-raised, thus artificial insemination is not a common practice in beef-herds, breeding usually occuring by natural mating, with several sources of potential error in pedigree assignment.

Many factors may contribute to pedigree errors in cattle, including mistakes in recording mating or insemination events, interchange of calves at birth and multiple sire- breeding groups (Christensen *et al.*, 1982). A retrospective assessment of the type of incompatibility occurring in parentage control may be useful in detecting the sources of errors committed in pedigree recording, and thus provide a basis for taking appropriate measures that may improve the situation (Visscher *et al.*, 2002, Weller *et al.*, 2004, Jiménez-Gamero *et al.*, 2006).

Microsatellite markers have been extensively used in individual identification and parentage control, with several advantages when compared with traditional genetic markers such as blood groups or proteins, since they are distributed in large numbers throughout the genome, have high levels of polymorphism, show co-dominant inheritance, their analysis is easily made automatic and several microsatellite loci can be analysed simultaneously (Cañón *et al.*, 2001). Parentage-control based on powerful genetic markers, such as microsatellites, can be achieved either by checking the compatibility of an offspring genotype with that of the alleged parent, or through pedigree assignment, *i.e.* by choosing the most likely parent from a group of potential ancestors (Van Eenennaam *et al.*, 2007), if breeding records are not available.

The objectives of this study were 1) to develop a panel of microsatellite markers useful for routine parentage-control in beef cattle produced under range conditions in Portugal and 2) apply this panel to a sample of registered calves with assigned parentage, but where there is a suspicion of error in recording possible parentage, in order to assess the level of errors in sire and dam-identification.

In the development stage of this study, hair samples were collected from 475 registered animals of the Charolais (CH, n = 153), Limousin (LI, n = 122) and Preta (PR, n = 200) breeds, and randomly sampled in 50, 11 and 4 herds, respectively. In the application stage, 1571 animals of these and other breeds were sampled, of which 1431 were potential parents kept in the DNA bank, and 140 were registered calves with recorded sire and dam, for which confirmation of parentage was requested since the reliability of pedigree recording was under suspicion.

A set of 10 microsatellite markers was selected, according to the recommendations of the Food and Agriculture Organization of the United Nations and the International Society of Animal Genetics, regarding genetic diversity studies and parentage control in cattle (FAO, 2004). The markers used were BM1824 (Bishop and Kappes, 1994), BM2113 (Bishop and Kappes, 1994), ETH10 (Toldo and Fries, 1993), ETH225 (Steffen and Eggen, 1993), INRA023 (Vaiman and Mercier, 1994), SPS115 (Moore and Byrne, 1994), TGLA53 (Kappes *et al.*, 1997), TGLA122 (Barendse and Armitage, 1994), TGLA126 (Kappes *et al.*, 1997) and TGLA227 (Kappes *et al.*, 1997). The microsatellite markers were grouped into one multiplex PCR reaction, and primers were labelled with fluorescent markers of three colours to distinguish between fragments of a similar size.

DNA was extracted from hair-roots with Chelex^®^ 100 (Bio-Rad) and proteinase-K (Qbiogen), as described by Walsh *et al.* (1991), and kept frozen at -18 °C until further processing. Amplification of target DNA was carried out by PCR, with 1 μL extracted DNA added to sterilized water, 2.5 μL of primers mixture at 0.2 pmol and the Qiagen Master Mix (containing Hotstart DNA Polymerase, buffer multiplex PCR with MgCl_2_ and dNTP mix), according to manufacturer's recommendations. Thermo-cyclers were programmed to start at 95 °C (15 min), followed by a series of 30 cycles, with denaturing at 94 °C (30 s), annealing at 57 °C (3 min) and extension at 72 °C (1 min), with a final elongation step of 30 min at 60 °C and ending at 4 °C.

The PCR products were submitted to fragments analysis by capillary electrophoresis, with an automated sequencer ABI310 (Applied Biosystems, Applera Europe B.V.), using the ROX^®^ size standard according to manufacturer's specifications. Results from capillary electrophoresis were read directly and interpreted with Genescan^®^ and Genotyper^®^ software, respectively.

In the development stage of the experiment, standard statistical procedures were used to assess the usefulness of the set of genetic markers selected for parentage control, based on information generated from the three breeds where implementation took place. The number of alleles per locus (n_a_) was obtained by direct counting, and the corresponding allele frequencies were used to calculate expected heterozygosity (H_e_) and the effective number of alleles per locus (n_e_), as described by Falconer and Mackay (1996) and Hartl and Clark (1997). The polymorphic information content (PIC) of a given locus was computed as in Botstein *et al.* (1980), while the probability of exclusion of a given locus in parentage testing (PE) and the combined probability of exclusion with a set of markers (CPE) were calculated according to Jamieson and Taylor (1997). Differences among breeds in PE by locus were tested by chi-square analysis, assuming that the expected number would be that corresponding to the mean PE for the three breeds.

Parentage testing was carried out by assessing compatibility between alleles present in a calf and those found in the assumed parents. As suggested by Luikart *et al.* (1999) and Weller *et al.* (2004), an assigned parent was excluded if its genotype was incompatible in two or more loci with that of the offspring, but parentage was not excluded if incompatibility occurred in only one locus.

All analyzed microsatellite markers showed amplification in one multiplex reaction carried out under the described experimental conditions, and the choice of colour labels warranted appropriate distinction of the different markers. Allele frequencies are graphically represented by locus and breed in Figure 1, and are available from the corresponding author upon request. Major differences among breeds in the distribution of alleles were observed, so that, for example, there was only one largely predominant allele in CH in the ETH10 locus, whereas for the PR breed, predominant alleles were found in INRA23, SPS115, TGLA122 and TGLA227. On the other hand, there was a wide spread of allele frequencies in all breeds for marker BM2113, this reflecting the high level of polymorphism of this microsatellite.

The total number of alleles found for the 10 microsatellite markers was 107, and polymorphisms in all loci were observed for the three breeds (Table 1). The overall mean n_a_ per locus was 8.20, with the highest value observed in the PR breed. On the other hand, when compared with the other two breeds, the CH had the lowest mean n_a_ and a smaller number of alleles per locus in all the loci except INRA23. Across breeds, the highest n_a_ was found for TGLA53 (10.67) and TGLA227 (10.00) loci, while the lowest mean was observed for BM1824 (5.67).

The n_e_ (which provides an indication of the number of alleles that would result in the observed genetic variability, if they all had the same frequency) differed widely between loci, ranging from about 2.9 (TGLA126 and SPS115) to about 6.1 (INRA23 and BM2113). Among breeds, the highest n_e_ was found in LI (4.81) and the lowest in PR (3.97). Large differences were detected between breeds for the different loci, so that in ETH10 only 1.28 effective alleles were found in CH, compared to about 4.2 in LI and 4.6 in PR. On the contrary, the highest n_e_ for INRA23 was observed in CH (10.14), with much lower values in LI (5.21) and PR (3.02).

The n_e_/n_a_ ratio indicates how well distributed the alleles are, relative to their number in a given locus-breed combination, so that a low ratio indicates the predominance of only a few alleles in a given locus. The n_e_/n_a_ ratio had a global mean of 0.54, with breed means ranging from 0.46 (PR) to 0.58 (LI). Among loci, the mean n_e_/n_a_ ratio ranged between 0.37 (SPS115) and 0.69 (BM2113). The extreme values for this ratio were both found in CH, with the lowest value for n_e_/n_a_ in ETH10 (0.21) and the highest in INRA23 (0.85). Overall, distribution was better in LI and CH than in PR, with the n_e_/n_a_ ratio being below 0.4 for six loci in PR, two in CH, and none in LI. When the mean ratio per locus was considered across breeds, the loci with the most unbalanced distribution were SPS115 and TGLA126, while those with a better spread were BM1824 and BM2113.

The mean H_e_ for the set of 10 microsatellites used was 0.733, ranging among breeds from 0.697 (CH) to 0.774 (LI). All the loci showed high levels of genetic variability, with heterozygosity ranging between 0.587 (ETH10) and 0.837 (BM2113). Nevertheless, the H_e_ by locus differed considerably among breeds, with estimates ranging between 0.221 (ETH10) and 0.901 (INRA23) in CH, 0.614 (TGLA126) and 0.838 (TGLA122) in LI, and 0.598 (SPS115) and 0.853 (BM2113) in PR.

The means for n_a_ and H_e_ indicate high levels of genetic diversity in the populations studied, and are within the range found in other Portuguese (Mateus *et al.*, 2004) and southern European (Cañón *et al.*, 2001) breeds of cattle, but are higher than in northern European (Kantanen *et al.*, 2000), French (Maudet *et al.*, 2002) and British (Wiener *et al.*, 2004) breeds. However, differences in genetic diversity between breed-loci combinations were important, with higher levels of heterogeneity in CH, which had the more extreme values for n_a_ (markers BM1824 and INRA23) and H_e_ (markers ETH10 and INRA23).

The overall mean PIC for all breeds and loci was 0.70, ranging between 0.67 (CH) and 0.74 (LI) among breeds, and between 0.56 (ETH10) and 0.82 (BM2113) among loci. For the different breed-locus combinations and with the exception of the ETH10 locus in CH, all PIC estimates were above 0.5, indicating that they are very useful for genetic diversity studies (Botstein *et al.*, 1980). As expected, there was close agreement between n_e_ and PIC for breed-locus combination (r = 0.83, p < 0.01), and in general microsatellites BM2113 and TGLA53 were the most informative loci.

PE corresponds to the probability that a random individual other than a true parent can be proven not to be the true parent of another randomly chosen individual, assuming that the population is in Hardy-Weinberg equilibrium (Van Eenennaam *et al.*, 2007). In our study, PE by locus-breed closely followed the pattern observed for PIC, with loci BM2113 and TGLA53 having the highest PE across the three breeds. Nevertheless, the usefulness of the various markers in parentage testing differed among breeds (p < 0.05) for five of the 10 loci analyzed (Table 1), so that the most useful marker for parentage testing was INRA23 in CH, TGLA53 in LI and BM2113 in PR. This indicates that, if a reduced number of markers is used in parentage testing, it may be appropriate to use a breed-specific set of markers, as a few are not very informative for some of the breeds (ETH10 in CH, TGLA126 in LI and SPS115 in PR).

However, a commercial service for pedigree validation would presumably have to be applied in several breeds, and the set of markers used here seems to have a high potential for serving that purpose, in spite of the fact that a few of the markers may be of limited usefulness in some of the breeds. The set of 10 markers resulted in a CPE by breed ranging between 0.9995 (PR) and 0.9999 (LI), thus confirming the very high potentiality of this marker-set for parentage testing in the group of breeds evaluated. Nevertheless, it should be recognized that, in practical situations, the discriminating power of a set of markers might be lower, if related individuals are used as breeders. In this case, true parents and their relatives have common alleles, and the ability to exclude a putative parent would require a larger set of markers to achieve the same reliability.

The cumulative CPE with an increasing number of microsatellite markers is shown in Figure 2 for the three breeds, with markers chosen in decreasing order according to their informativeness in the CH breed. It is clear from Figure 2 that with six markers, CPE is above 0.99 for all the three breeds, and a marginal improvement is obtained when additional markers are considered after this point. It is also apparent that CPE is lower for the PR breed, which is partly due to the fact that the sequence of markers chosen for the graph was the most potential for CH, whereas the decreasing order of markers would be different for PR.

Our marker-set was very similar to that used by Visscher *et al.* (2002), who used in addition microsatellite ETH3, discarding TGLA53 due to inconsistent results. When compared with our results, these authors found a slightly lower CPE with their marker-set when applied to British Holstein cattle. Nevertheless, Heyen *et al.* (1997), with a different set of 11 markers applied to American Holstein, reported a CPE similar to ours.

The set of 10 microsatellite markers was used for parentage testing in 140 calves and their assumed parents, in herds kept under extensive conditions, where pedigree recording was suspected to be unreliable, and incompatibility was declared if disagreement between parent and offspring occurred in two or more loci. The results of these analyses are summarized in Table 2, indicating that only about 76% of the calves were compatible with their assigned parents, while nearly 2% were incompatible with both of the parents, 7% incompatible with the sire and 14% incompatible with the dam. This suggests that most pedigree errors occur as a result of inadequate recording of calving events, and to a lesser extent, to incorrect assignment of the sire, possibly due to situations of multiple sires in a breeding group.

The rate of paternity-misidentification in the Holstein breed has been reported to be 13% in Germany (Gelderman *et al.*, 1986), 12% in the Netherlands (Bovenhuis and van Arendonk, 1991), 12% in Israel (Weller *et al.*, 2004), 12 to 15% in New Zealand (Spelman, 2002), and 10% in the United Kingdom (Visscher *et al.*, 2002). Misidentification rates for beef-cattle breeds kept in extensive production systems have not been very often reported, but are likely to be higher than in dairy cattle, due to the limited use of artificial insemination. For example, Baron *et al.* (2002) have reported rates of error in paternity identification of 36% for the Gir breed in Brazil, and methods have been proposed to optimize paternity-identification in beef cattle breeds kept under range conditions (Van Eenennaam *et al.*, 2007, Gomez-Raya *et al.*, 2008).

In our analysis, we chose to consider incompatibility of pedigrees if parent and offspring differed in two or more loci, as suggested by Heyen *et al.* (1997), Luikart *et al.* (1999) and Weller *et al.* (2004), to account for occasional genotyping errors, for the presence of null alleles (Petersen and Bendixen, 2000) and for the high mutation rate expected in microsatellites (Ellegren, 1995, Luikart *et al.*, 1999). In any case, in our analysis only one animal showed incompatibility with the parents in one locus alone, and the mean number of incompatible loci between offspring and parents ranged between 3.6 for sires and 5.2 for dams. Thus, the conservative approach used here of considering a minimum of two markers as the criterion for rejecting compatibility of the parents seems appropriate, as it minimizes the possibility of wrongly rejecting a true parent.

In conclusion, the set of 10 microsatellite markers tested in this study proved to be easy to implement in one multiplex reaction, and the degree of polymorphism observed in three different cattle breeds confirms the usefulness of this panel for parentage testing, even though the value of individual markers depended on the breed under consideration. The application of the marker panel in pedigree checking in a group of commercial beef calves, where suspicion of error in recording pedigree existed, reveals that the level of misidentification gives rise to some concern and that steps must be taken to improve the quality of records, especially at the time of calving.

**Figure 1:**
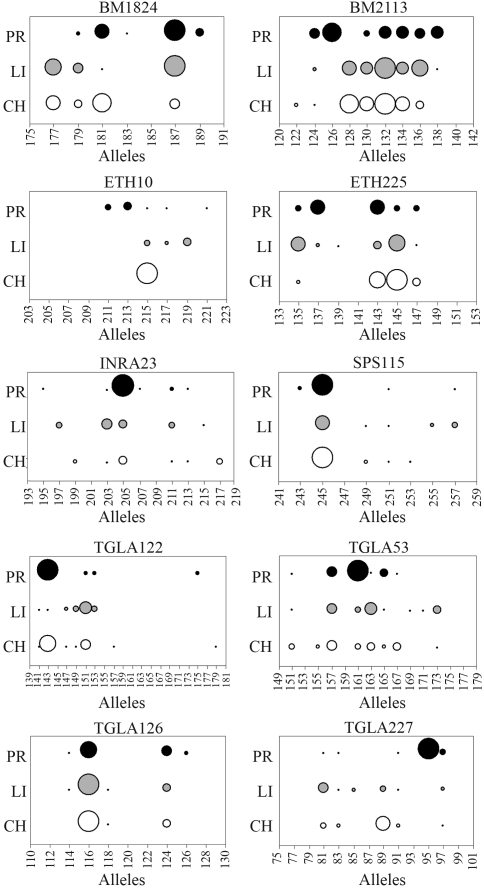
Allele frequencies by locus-breed combination, with bubble size proportional to frequency by breed (CH = Charolais, LI = Limousin, PR = Preta).

**Figure 2:**
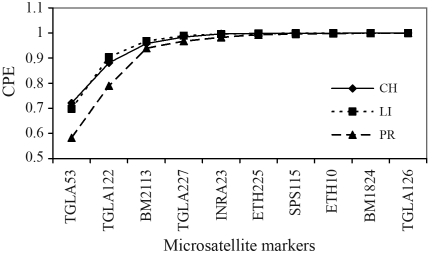
Combined probability of exclusion (CPE) in parentage testing with increasing number of microsatellite markers, by breed (CH = Charolais, LI = Limousin, PR = Preta).

## Figures and Tables

**Table 1:** Total number of alleles (n_a_), effective number of alleles (n_e_), n_e_/n_a_ ratio, expected heterozygosity (H_e_), polymorphic information content (PIC) and probability of exclusion (PE) by breed and locus, and significance of difference among breeds in the probability of exclusion by locus [p (χ^2^)].

		Microsatellite loci										
	Breed	BM1824	BM2113	ETH10	ETH225	INRA23	SPS115	TGLA122	TGLA53	TGLA126	TGLA227	Global
n_a_	CH	5	8	6	6	12	7	6	10	7	10	7.70
	LI	6	8	7	8	9	8	8	11	6	11	8.20
	PR	6	11	7	9	8	8	9	11	9	9	8.70
	Mean	5.67	9	6.67	7.67	9.67	7.67	7.67	10.67	7.33	10	8.20

n_e_	CH	3.83	5.68	1.28	3.47	10.14	2.26	4.10	6.96	2.61	4.69	4.50
	LI	3.21	5.99	4.16	4.60	5.21	3.86	6.16	6.42	2.59	5.89	4.81
	PR	3.94	6.80	4.57	4.90	3.02	2.49	3.31	4.31	3.38	2.96	3.97
	Mean	3.66	6.16	3.34	4.32	6.12	2.87	4.52	5.90	2.86	4.51	4.43

n_e_/n_a_	CH	0.77	0.71	0.21	0.58	0.85	0.32	0.68	0.70	0.37	0.47	0.57
	LI	0.54	0.75	0.59	0.57	0.58	0.48	0.77	0.58	0.43	0.54	0.58
	PR	0.66	0.62	0.65	0.54	0.38	0.31	0.37	0.39	0.38	0.33	0.46
	Mean	0.66	0.69	0.48	0.56	0.60	0.37	0.61	0.56	0.39	0.45	0.54

H_e_	CH	0.739	0.824	0.221	0.712	0.901	0.557	0.756	0.856	0.617	0.787	0.697
	LI	0.689	0.833	0.760	0.783	0.808	0.741	0.838	0.844	0.614	0.830	0.774
	PR	0.746	0.853	0.781	0.796	0.669	0.598	0.698	0.768	0.704	0.662	0.728
	Mean	0.725	0.837	0.587	0.764	0.793	0.632	0.764	0.823	0.645	0.760	0.733

PIC	CH	0.69	0.80	0.22	0.66	0.85	0.53	0.74	0.85	0.57	0.79	0.67
	LI	0.63	0.81	0.72	0.75	0.78	0.70	0.82	0.83	0.57	0.81	0.74
	PR	0.71	0.84	0.75	0.76	0.64	0.56	0.67	0.74	0.67	0.64	0.70
	Mean	0.68	0.82	0.56	0.72	0.76	0.60	0.74	0.81	0.60	0.75	0.70

PE	CH	0.498	0.650	0.121	0.467	0.802	0.351	0.573	0.721	0.377	0.608	0.9997
	LI	0.425	0.665	0.533	0.585	0.625	0.539	0.681	0.698	0.384	0.664	0.9999
	PR	0.524	0.711	0.583	0.598	0.471	0.380	0.495	0.574	0.386	0.467	0.9995
	Mean	0.482	0.675	0.412	0.55	0.633	0.423	0.583	0.664	0.382	0.580	0.9997

p (χ^2^)		ns	ns	**	ns	**	**	*	ns	ns	*	ns

**Table 2:** Proportion of calves with correct and incorrect assignment of sire and dam, and mean number of incompatible loci.

	Proportion of calves	Mean number of incompatible loci
Correct assignment of sire and dam	0.764	-
Incorrect assignment of both sire and dam	0.021	3.8
Incorrect assignment of sire	0.071	3.6
Incorrect assignment of dam	0.136	5.2
